# Supplementary material for: Breaking the cycle: Systematic review of perinatal interventions for parents at risk of child removal
Source: PLoS One. 2025 Nov 26;20(11):e0337711. doi: 10.1371/journal.pone.0337711 (PMC12654914; doi:10.1371/journal.pone.0337711)
Supplement: S2 Appendix — (DOCX) [file pone.0337711.s002.docx]

**Search Terms**

“Baby Remov*” Or “Infant Entry” Or “Child Remov*” Or “Infant Remov*” Or “Remov* At Birth” or “Recurrent Care” Or “Repeat Care Proceedings”

And

Intervention Or Prevent* Or “ Systemic Solution” Or Reduc* Or Servic*

And

Parent* Or Famil*Or Birth Mother Or Mother Or Father Or Maternal OR Paternal

And

"Family Court" Or "Child Protective Service*" Or "Care Order" Or "Family Justice" Or "Local Authority" Or "Care Proceeding" Or "Child Welfare Case*" Or "Child Protection"

( “Baby Remov*” Or “Infant Entry” Or “Child Remov*” Or “Infant Remov*” Or “Remov* At Birth” or “Recurrent Care” Or “Repeat Care Proceedings” ) AND ( Intervention Or Prevent* Or “ Systemic Solution” Or Reduc* Or Servic* ) AND ( Parent* Or Famil*Or Birth Mother Or Mother Or Father Or Maternal OR Paternal ) AND ( "Family Court" Or "Child Protective Service*" Or "Care Order" Or "Family Justice" Or "Local Authority" Or "Care Proceeding" Or "Child Welfare Case*" Or "Child Protection" )

**Furthermore, we will hand-search relevant funding body and charity websites, including**:

- The Nuffield Foundation [www.nuffieldfoundation.org](http://www.nuffieldfoundation.org) ;
- Research in Practice UK <https://www.researchinpractice.org.uk/>
- Family Rights Group <https://www.frg.org.uk/>
- Children and Family Court Advisory Service (Cafcass) <https://www.cafcass.gov.uk/>
- Community of Practice - UK research in practice
- Australian Institute of Family Studies
- California Evidence-Based Clearinghouse for Child Welfare
- Chapin Hall at the University of Chicago
- Washington State Institute for Public Policy
- Office of Planning, Research, and Evaluation, US Administration for Children and Families
- Foundations / What Works for Children's Social Care
- Rees Centre
- CASCADE: Children's Social Care Research and Development Centre(<https://cascadewales.org/Gur-research>!)
- CELCIS: Centre for Excellence for Children's Care and Protection (https:J/www.celcis.orgJour-work/research)
