# Supplementary material for: Breaking the cycle: Systematic review of perinatal interventions for parents at risk of child removal
Source: PLoS One. 2025 Nov 26;20(11):e0337711. doi: 10.1371/journal.pone.0337711 (PMC12654914; doi:10.1371/journal.pone.0337711)
Supplement: S3 File — (DOCX) [file pone.0337711.s003.docx]

Risk of Bias using MMAT tool

|  | **SCREENING QUESTIONS** | | **3. NON-RANDOMIZED STUDIES** | | | | | **5. MIXED METHODS STUDIES** | | | | |
| --- | --- | --- | --- | --- | --- | --- | --- | --- | --- | --- | --- | --- |
| Citation | S1 | S2. | 3.1 | 3.2 | 3.3 | 3.4 | 3.5 | 5.1 | 5.2 | 5.3 | 5.4 | 5.5 |
| Cox, P., McPherson, S., Mason, C., Ryan, M., & Baxter, V. (2020). Reducing recurrent care proceedings: Building a local evidence base in England. Societies, 10(4), 88. | Yes | Yes |  |  |  |  |  | Yes | Yes | Yes | No | Yes |
| Harnett, P. H., Barlow, J., Coe, C., Newbold, C., & Dawe, S. (2018). Assessing capacity to change in high‐risk pregnant women: a pilot study. Child abuse review, 27(1), 72-84. | Yes | Yes |  |  |  |  |  | Yes | Yes | Yes | No | Yes |
| O'Dea, B., Roe, Y., Gao, Y., Kruske, S., Nelson, C., Hickey, S., ... & Kildea, S. (2024). Breaking the cycle: Effect of a multi-agency maternity service redesign on reducing the over-representation of Aboriginal and Torres Strait Islander newborns in out-of-home care: A prospective, non-randomised, intervention study in urban Australia. Child Abuse & Neglect, 149, 106664. | Yes | Yes | Yes | Yes | Yes | Yes | Yes |  |  |  |  |  |
| Taplin, S. (2017). Prenatal reporting to child protection: characteristics and service responses in one Australian jurisdiction. *Child Abuse & Neglect*, *65*, 68-76. | Yes | Yes | Yes | Yes | Yes | Yes | Yes |  |  |  |  |  |
| Segal, L., Nguyen, H., Gent, D., Hampton, C., & Boffa, J. (2018). Child protection outcomes of the Australian Nurse Family Partnership Program for Aboriginal infants and their mothers in Central Australia. *PLoS One*, *13*(12), e0208764. | Yes | Yes | Yes | Yes | Yes | Yes | Yes |  |  |  |  |  |
| Shaw, M. (2021). A Proof-of-Concept Pilot for an Intervention with Pregnant Mothers Who Have Had Children Removed by the State: The ‘Early Family Drug and Alcohol Court Model’. *Societies*, *11*(1), 8. | Yes | Yes |  |  |  |  |  |  | Yes | Yes | Yes | Can’t tell |
