# Supplementary material for: Breaking the cycle: Systematic review of perinatal interventions for parents at risk of child removal
Source: PLoS One. 2025 Nov 26;20(11):e0337711. doi: 10.1371/journal.pone.0337711 (PMC12654914; doi:10.1371/journal.pone.0337711)
Supplement: Attachment — Submitted filename: PONE-D-25-19264 Reviewer Recommendations.docx [file pone.0337711.s004.docx]

***Breaking the Cycle: Systematic Review of Perinatal Interventions for Parents with Histories of Child Removal Systematic Review: Preventing Recurrent Infant Removals***

**PLOSOne Reviewer Rcommendation and Comments for Manuscript Number PONE-D-25-10264: Accept with Major Revisions**

This paper describes a systematic review of perinatal interventions for parents with histories of child removal, a significant topic that warrants further research. Overall, the topic is introduced well with relevant literature providing a strong rationale for the study. The methods of choice fit well with the stated research questions and overall appear to be conducted appropriately. Despite the significant findings from the review, the reporting of results appears inconsistent and hard to follow, leading to a weak discussion with clear conclusions and useful recommendations for future research and service design. Please see below specific recommendations that need improvement so the paper can make the positive contribution to the literature that is warranted.

1. Overall formatting, referencing, and punctuation needs review, to address many instances of missed spaces after or before a citation, ensuring citations are all represented in-text Vancouver style, and typographical errors throughout. Also need greater consistency of past/present tense language, particularly in the methodology and results sections.
2. Throughout the review there is reference to women and parents, but no specific discussion about fathers. Given the additional barriers and complexities associated with the population, can this please be addressed and either: include more specific details about men in the studies reviews, or rationale for excluding fathers from the review?
3. Methodology 2.0: What was the PROSPERO registration number?
4. Methodology 2.1: Can the search terms be clearly defined, with appropriate and consistent use of quotation marks, as represented in S2 attachment.
5. Methodology Figure 1: Ensure text has correct punctuation and capitalisation, and all lines are connecting to relevant boxes in the flow chart
6. Methodology 2.2: It would be clearer for the reader if eligibility and exclusion criteria were presented in dot point form. The comment that abstracts, conference proceedings.... grey literature are excluded, would be more clearly referenced as exclusion criteria.
7. Methodology 2.3: Needs significant review of language, use of past/present tense, and sentence structure.
8. Results: Needs significant review to improve clarity of expression, use of formal language and concise reporting. In particular, use of tables needs refining, consider formatting, use of language and overall presentation. E.g. Tables 3-6 are not clear what they are indicating and seem to use ticks across them for different reasons. Please consider representing this more clearly, such as with footnotes below the table to aid the reader. Subheads are also inconsistent, e.g. 3.5.1. speaks generally of enablers compared to 3.5.2-4 each represent a specific barrier. As the reporting of results appears combined with discussion points throughout, consider whether this section would be more accurately titled "Results/Discussion", or if the discussion points can be moved to the appropriate section.
9. Discussion: As above, this section seems to repeat the main results. Consider whether this could be more concisely presented a discussion of overall themes (as it seems to be towards the second half of the section), or more clearly defined responses to the main research questions.
10. Attachment S3: There are only 5 studies represented in the Risk of Bias MMAT tool, yet the review included 6.
