# Supplementary material for: Breaking the cycle: Systematic review of perinatal interventions for parents at risk of child removal
Source: PLoS One. 2025 Nov 26;20(11):e0337711. doi: 10.1371/journal.pone.0337711 (PMC12654914; doi:10.1371/journal.pone.0337711)
Supplement: Attachment — Submitted filename: Reviewer Feedback.docx [file pone.0337711.s005.docx]

## PLOS One: PONE-D-25-19264

## Breaking the Cycle: Systematic Review of Perinatal Interventions for Parents with Histories of Child Removal Systematic Review: Preventing Recurrent Infant Removals

**Reviewer Comments:**

This is an interesting and meaningful systematic review which considers several elements of the effectiveness of interventions designed to prevent infant removals.

This paper would benefit from an overall review and revision to strengthen the written expression and structure throughout. The authors may wish to review the appropriateness of information presented in each section, and if it is better suited to other sections within the paper, to avoid redundancy.

Further feedback has been provided in relation to each section of the paper to help guide authors in their revision.

Overarching Comments:

It is not clear throughout the paper if this review looks at interventions aimed at preventing *recurrent* infant removals (i.e., where parents have experienced previous infant removal) or infant removals in general. From the methods section and eligibility criteria, it appears to be the latter (interventions aimed at preventing infant removals in general, as there is no criteria based on previous removal experience). If this is the case, the authors may consider removing emphasis on recurrent infant removals, as this confuses the intent and target population of the interventions.

The authors may consider more consistently referring to 'infant removals' (as opposed to baby removals) throughout the paper.

It could be individual style or preference, but I encourage the authors to be mindful of overusing hyphens in any discussion throughout the paper.

Introduction:

The introduction section would benefit from more detailed discussion on the impact of infant removals, specifically, (as opposed to child removal broadly), including long-term impacts on parents, infant etc. as infant removals present their own unique challenges and psychosocial, cultural, and socioeconomic impacts for families.

The introduction section would also benefit from some data demonstrating the rate of infant removals across the contexts of focus for the review, including data for Indigenous populations specifically, considering two of the included studies in the review focus on Indigenous mothers.

Paragraph 2, Page 5 - This paragraph could be strengthened with a more detailed overview of early intervention service delivery (e.g., what types of support are being provided) and the elements which are thought to be important in effective early intervention strategies (e.g., see Keddell et al., 2023).

Objectives section - Rationales for incl./excl. criteria to be moved to eligibility criteria section. Suggest moving the definitions and descriptions of recurrent care interventions and care proceedings into the introduction, where these concepts can be introduced to the reader appropriately.

Methodology:

Paragraph 2, Page 8 - 'We conducted a systematic review to help us understand the interventions that prevent...' - suggest rewording 'us' to the wider academic audience - (e.g., it is also for the field to obtain a better understanding).

'A meta-analysis was not deemed possible based on preliminary literature search and evidence from literature' - While I appreciate grounds for not conducting a meta-analysis for this synthesis of literature, could the authors clarify this justification further for the reader (e.g., evidence was too heterogeneous?).

Paragraph 1, Page 10 - Could the authors briefly describe the child protection system orientation which is shared across the contexts mentioned (in relation to criteria ii).

Paragraph 1, Page 10 - Minor correction - these are three distinct criteria (in relation to criteria iii).

Paragraph 1, Page 10 – As per earlier comment, there is no mention in the eligibility criteria of parents previous removal history (i.e., if interventions were targeted towards parents who had a previous experience of infant/child removal). As this paper aims to investigate the effectiveness of perinatal interventions designed to prevent *recurrent* infant removals, it is unclear if the interventions reviewed are effective at preventing recurrent removals, or removals in general. Please clarify this in the eligibility criteria and how it was ensured interventions were targeted towards preventing recurrent infant removals, specifically.

Paragraph 1, Page 11 - Could the authors comment on or provide the result of interrater consistency/agreement?

Paragraph 2, Page 11 - Could the authors briefly describe the difference between descriptive and thematic analysis, if distinguishing between the two for different research questions?

Results:

Paragraph 1, Page 13 - Could the authors clarify how at risk of child removal was defined in these studies (e.g., received one or more pre-birth CP notifications, or, previous history of child removal?)

Paragraph 2, Page 13 - Could the authors describe what they're referring to as 'program data' here?

Paragraph 1, Page 14 - It would be beneficial to see further description here of the policy reform intervention, and what exactly this entailed/how it was implemented.

Paragraph 2, Page 14 - Quick correction: interventions*

Paragraph 2, Page 15 - The finding in relation to reference (39), demonstrating those in the intervention group to have increased rates of infant removal compared to the control group is very interesting. I would be interested in commentary or some insight to this in the discussion section.

Paragraph 1, Page 23 – Indigenous needs a capital ‘I’

It would be great if the authors included some further descriptions of the culturally safe support strategies implemented in some of the interventions and found to have a positive effective/be an enabler.

Discussion:

Although I appreciate the reviews’ focus of intervention effectiveness at preventing infant removals through supporting parents in minimising child protection risks, it would be good to see some acknowledgement in the discussion of the issue of heightened surveillance and bias in child protection practices and interventions, which can often discriminate against and are inequitable for certain minority or racial groups.

Acknowledgements:

Please include an acknowledgements section prior to the references, as per the journal’s requirements (<https://journals.plos.org/plosone/s/submission-guidelines>)
