# Supplementary material for: Breaking the cycle: Systematic review of perinatal interventions for parents at risk of child removal
Source: PLoS One. 2025 Nov 26;20(11):e0337711. doi: 10.1371/journal.pone.0337711 (PMC12654914; doi:10.1371/journal.pone.0337711)
Supplement: Attachment — Submitted filename: Response to Reviewers.docx [file pone.0337711.s006.docx]

**Response to Reviewers**

**Reviewer Comments:**

This is an interesting and meaningful systematic review which considers several elements of the effectiveness of interventions designed to prevent infant removals.

Thank you very much for your thoughtful and constructive feedback on the manuscript. We sincerely appreciate your	comments	regarding the introduction and methodology of the study.

This paper would benefit from an overall review and revision to strengthen the written expression and structure throughout. The authors may wish to review the appropriateness of information presented in each section, and if it is better suited to other sections within the paper, to avoid redundancy. Further feedback has been provided in relation to each section of the paper to help guide authors in their revision.

In light of your comments, the manuscript has been reviewed. The written structure has been addressed. For example. The result and discussion has been restructured and text about quality of Evidence is now put together as a subsection

Overarching Comments:

It is not clear throughout the paper if this review looks at interventions aimed at preventing *recurrent* infant removals (i.e., where parents have experienced previous infant removal) or infant removals in general. From the methods section and eligibility criteria, it appears to be the latter (interventions aimed at preventing infant removals in general, as there is no criteria based on previous removal experience).- If this is the case, the authors may consider removing emphasis on recurrent infant removals, as this confuses the intent and target population of the interventions.

The authors have removed the emphasis on recurrent infant removals and make it infant removal.

However, we do indicate that recurrent removal contributes significantly to the rate of incidence removal. This can be found in paragraph 2 of page 4 as

*“Research shows that recurrent care proceedings account for a significant portion of infant removals across countries, with first-time mothers in England, Indigenous groups in Australia, and Black families in the USA identified as particularly affected populations (5-7). Recurrent care proceedings includes “a cycle of returning to court and having subsequent children removed”(8). An Australian cohort study of 1,629 mothers (2000–2018) found that 20.3% of biological parents who had an infant removed by court order experienced repeat removals,* *with Indigenous groups representing the largest proportion in national infant removal statistics(9, 10). In Scotland, Wales and England respectively at least twenty percent of mothers who experience infant removal , were first time mothers (11, 12).In England, about 25% of mothers with a prior court-ordered infant removal subsequently faced repeat care proceeding within 10years (13, 14). In England and Wales respectively, half of mothers involved in care proceedings were subject to repeat care proceedings within eight years and five years respectively (15). 92% of Scottish mothers with older children in care proceedings, had previously had at least one child removed(16). Studies in the U.S. show that birth mothers with a previous child removed have significantly higher odds of experiencing another removal (17), with black children over represented at 20% in 2021(18). “*

The authors may consistently referring to 'infant removals' (as opposed to baby removals) throughout the paper.

*This has been changed and there is a consistent use of the term infant removal in the text.*

It could be individual style or preference, but I encourage the authors to be mindful of overusing hyphens in any discussion throughout the paper.-

*This is noted and has been corrected*

Introduction:

The introduction section would benefit from more detailed discussion on the impact of infant removals, specifically, (as opposed to child removal broadly), including long-term impacts on parents, infant etc. as infant removals present their own unique challenges and psychosocial, cultural, and socioeconomic impacts for families.

We thank the reviewer for highlighting this important point. We agree that infant removals have unique implications distinct from broader child removal and warrant specific discussion. In response, the Introduction has been tailored to include literature that are specific on the psychosocial, cultural, and socioeconomic impacts of infant removals, as well as long-term consequences for both infants and parents.

The introduction section would also benefit from some data demonstrating the rate of infant removals across the contexts of focus for the review, including data for Indigenous populations specifically, considering two of the included studies in the review focus on Indigenous mothers.

Thank you for this valuable suggestion. We agree that including data on the rate of infant removals across the contexts of focus would strengthen the introduction and provide important context. We have now incorporated relevant statistics highlighting infant removal rates, including data specific to Indigenous populations, to reflect the focus of the studies included in our review. This addition clarifies the scope and significance of the issue for the populations under study. It is as shown below

*Emerging evidence shows a significant portion of child removals involve infants (1). In Australia, infants (children under 1 year old) represent the largest proportion of child removals, with a removal rate of 6.5 percent 1,000 which is significantly higher than the rate of 2.0 per 1,000 observed in other age groups between 2023 and 2024(2). Similarly in England, 70 per 10000 children under one year old were in care proceeding in comparison to 19 per 10,000 children aged between one and four years old(3). It can be found on Page 4*

Paragraph 2, Page 5 - This paragraph could be strengthened with a more detailed overview of early intervention service delivery (e.g., what types of support are being provided) and the elements which are thought to be important in effective early intervention strategies (e.g., see Keddell et al., 2023).

This has been addressed and the	text now includes	a short description of early service intervention as shown below and can be found on page 6

*“There is growing but still limited evidence that intensive, relational, and tailored interventions, that combines practical, therapeutic, emotional support and advocacy, with a focus on reducing parental stress and drawing on parents’ commitment to their children can be effective in reducing the rate of infant removal”*

Objectives section - Rationales for incl./excl. criteria to be moved to eligibility criteria section. Suggest moving the definitions and descriptions of recurrent care interventions and care proceedings into the introduction, where these concepts can be introduced to the reader appropriately.

The definitions has been moved to the introduction first paragraph and rationales have been moved to the eligibity criteria section

Methodology:

Paragraph 2, Page 8 - 'We conducted a systematic review to help us understand the interventions that prevent...' - suggest rewording 'us' to the wider academic audience - (e.g., it is also for the field to obtain a better understanding).

We have now reworded the sentence. Please see page 9, lines 183-185: We conducted this systematic review to investigate the effectiveness of perinatal interventions designed to prevent infant removals and recurrent care proceedings, including the context in which they work, focusing on interventions that begin during pregnancy.

'A meta-analysis was not deemed possible based on preliminary literature search and evidence from literature' - While I appreciate grounds for not conducting a meta-analysis for this synthesis of literature, could the authors clarify this justification further for the reader (e.g., evidence was too heterogeneous?).

We have now strengthened the justification for not conducting meta-analysis. Please see page 13, lines 289-290: We did not conduct a meta-analysis given the substantial heterogeneity in the results of individual studies which could undermine the validity of the pooled results (36).

Paragraph 1, Page 10 - Could the authors briefly describe the child protection system orientation which is shared across the contexts mentioned (in relation to criteria ii).

A description of the chid protection orientation and similarity across the context is included in the criteria. It is now numbered as criteria iii

*“These countries share a child protection orientation that is broadly comparable to the UK model, in which the state holds statutory powers to intervene in cases of child maltreatment, including the authority to remove children from parental care through court proceedings. These systems are primarily child-focused, operate under a legal framework that prioritises the child’s safety and welfare, and allow for both voluntary and involuntary out-of-home placements. They also share features such as adversarial or quasi-adversarial court processes, mandatory reporting duties, and a focus on preventing re-entry into care, making them relevant for comparison with the UK context of high rates of infant removal and repeat removals”* page

Paragraph 1, Page 10 - Minor correction - these are three distinct criteria (in relation to criteria iii).

The criteria has been split into 2

Paragraph 1, Page 10 – As per earlier comment, there is no mention in the eligibility criteria of parents previous removal history (i.e., if interventions were targeted towards parents who had a previous experience of infant/child removal). As this paper aims to investigate the effectiveness of perinatal interventions designed to prevent recurrent infant removals, it is unclear if the interventions reviewed are effective at preventing recurrent removals, or removals in general. Please clarify this in the eligibility criteria and how it was ensured interventions were targeted towards preventing recurrent infant removals, specifically.

The focus of the intervention is not on recurrent care only, but on general infant removal. We have included a statement about this and mentioned how the interventions are targeted towards prevent infant removal. The criteria has specified this.

*“It includes an intervention aimed at preventing child removal for pregnant mothers at risk of child removal. “ page 11*

The relevance of recurrent care removal to general infant removal is discussed in the introduction.

Paragraph 1, Page 11 - Could the authors comment on or provide the result of interrater consistency/agreement?

We have comment about interrater agreement as *“Interrater agreement during screening was moderate, with a proportionate agreement of 71% and a Cohen’s kappa of 0.39, indicating fair agreement (47).” Page 12*

Paragraph 2, Page 11 - Could the authors briefly describe the difference between descriptive and thematic analysis, if distinguishing between the two for different research questions?

We thank the reviewer for this helpful comment. We agree that it is important to clarify the distinction between descriptive and thematic analysis in relation to our research questions.The manuscript now includes

*“For research question one, which focused on identifying interventions and policies that reduce infant removals, we used descriptive analysis to summarise and categorise the types of interventions reported across studies. This approach allowed us to map the range, and characteristics of interventions in a structured way. For research questions two to five, which explored effectiveness across different parent groups, common elements of interventions, implementation factors, and parents’ perspectives, we used thematic analysis. (46). This approach enabled us to synthesise qualitative and mixed-methods findings, identify recurring patterns, and generate overarching themes across studies. We then provide a narrative of all findings in alignment with our study objectives. Data was synthesised per each research objective. We did not conduct a meta-analysis given the substantial heterogeneity in the results of individual studies which could undermine the validity of the pooled results”*page 13

Results:

Paragraph 1, Page 13 - Could the authors clarify how at risk of child removal was defined in these studies (e.g., received one or more pre-birth CP notifications, or, previous history of child removal?)

We have included how risk of infant removal was defined in the studies and is as shown below

*“All studies included mothers that were at risk of child removal based on previous child removal (53, 54), or referral to child protection service (30, 52), vulnerable Indigenous (first nations) mothers (50, 51)”* page 15, line 324-326

Paragraph 2, Page 13 - Could the authors describe what they're referring to as 'program data' here?

This is now included and stated as

“Data sources included program data collected during the implementation of the intervention (50, 53), administrative datasets (30, 51) and a combination of program data and validated measures (31, 52).” Page 15, line 327

Paragraph 1, Page 14 - It would be beneficial to see further description here of the policy reform intervention, and what exactly this entailed/how it was implemented.

We appreciate this suggestion and have now provided a clearer description of the prenatal reporting policy and its implementation.It is included as shown below

“The prenatal reporting policy includes the notification of child protection services during pregnancy about an unborn child that is considered at risk, with the aim of preventing harm after birth or having the baby taken into care and it offered voluntary assessments, child protection assessment at birth, referral to support services, and case work support for the coordination of services (30)” page 16 line 345

Paragraph 2, Page 14 - Quick correction: interventions*

Thanks for the observation, this is corrected

Paragraph 2, Page 15 - The finding in relation to reference (39), demonstrating those in the intervention group to have increased rates of infant removal compared to the control group is very interesting. I would be interested in commentary or some insight to this in the discussion section.

We have include a commentary about this into the manuscript

“The authors suggests that this likely reflects a more timely and informed decision making by practitioners, and action to prevent longer term harm. *” page 18,line 386*

Paragraph 1, Page 23 – Indigenous needs a capital ‘I’

*Thanks , this is corrected*

It would be great if the authors included some further descriptions of the culturally safe support strategies implemented in some of the interventions and found to have a positive effective/be an enabler.

We thank the reviewer for this suggestion. We have now included additional details of the culturally safe strategies within the BiOC model, highlighting Indigenous leadership and governance, continuity of care through midwives and First Nations Family Support Workers, staff training in cultural integrity frameworks, and community hubs that promote connection, support, and engagement with Elders as shown below

*“**BiOC’s multi-agency partnership model, led by Indigenous organisations, successfully reduced newborn removals through culturally safe, multi sectoral support to women. Key characteristics included Indigenous leadership and governance, continuity of care provided by dedicated midwives and Indigenous family support workers, integration of cultural integrity frameworks into staff training, and the creation of community hubs that fostered connection, peer support, and engagement with indigenous elders(51)”*page 28 line 533

Discussion:

Although I appreciate the reviews’ focus of intervention effectiveness at preventing infant removals through supporting parents in minimising child protection risks, it would be good to see some acknowledgement in the discussion of the issue of heightened surveillance and bias in child protection practices and interventions, which can often discriminate against and are inequitable for certain minority or racial groups.

This now included in the Conclusion and impication and written as shown below

*“Although the review focused on intervention effectiveness in infant removals, it is important to acknowledge critiques that heightened surveillance within child protection systems can itself have harmful consequences, particularly when disproportionately applied to minority and marginalised groups. Such practices risk reinforcing stigma and inequities, with Indigenous families and other racial or cultural minorities often subject to greater scrutiny and intervention despite systemic disadvantage. Addressing these concerns requires not only culturally safe, community-led services, but also broader reforms that actively confront bias and promote fairness in child protection decision-making.”page 37, line 677*

Acknowledgements:

Please include an acknowledgements section prior to the references, as per the journal’s requirements (<https://journals.plos.org/plosone/s/submission-guidelines>) -

*This is now included as shown below*

*“****Acknowledgements***
*We would like to acknowledge the contributions of the authors of the studies included in this review. No external funding or assistance was received for this work.”*

***Breaking the Cycle: Systematic Review of Perinatal Interventions for Parents with Histories of Child Removal Systematic Review: Preventing Recurrent Infant Removals***

**PLOSOne Reviewer Rcommendation and Comments for Manuscript Number PONE-D-25-10264: Accept with Major Revisions**

This paper describes a systematic review of perinatal interventions for parents with histories of child removal, a significant topic that warrants further research. Overall, the topic is introduced well with relevant literature providing a strong rationale for the study. The methods of choice fit well with the stated research questions and overall appear to be conducted appropriately. Despite the significant findings from the review, the reporting of results appears inconsistent and hard to follow, leading to a weak discussion with clear conclusions and useful recommendations for future research and service design. Please see below specific recommendations that need improvement so the paper can make the positive contribution to the literature that is warranted.

1. Overall formatting, referencing, and punctuation needs review, to address many instances of missed spaces after or before a citation, ensuring citations are all represented in-text Vancouver style, and typographical errors throughout. Also need greater consistency of past/present tense language, particularly in the methodology and results sections.

Thanks for the comments. The manuscript has been thoroughly proofread ensuring there is consistency throughout, avoiding double spaces, ensuring there is a space before citation, correcting minor grammatical errors and ensuring the use of the past tense in appropriate places in the methodology and results section.

2. Throughout the review there is reference to women and parents, but no specific discussion about fathers. Given the additional barriers and complexities associated with the population, can this please be addressed and either: include more specific details about men in the studies reviews, or rationale for excluding fathers from the review?

We have include a statement to state that no study reported on fathers engagement in the description of participants and in the imitations. It as shown below

*“None of the included study reports on any intervention with fathers or engagement with fathers. “ page 15, line 319*

*“In addition, the review found little evidence regarding parental perspectives, fathers’ engagement …”page 38, line 693*

3. Methodology 2.0: What was the PROSPERO registration number?

This is now included in the text

*“The review protocol was registered in the Prospective Register of Systematic Reviews (PROSPERO), registration number CRD42024561692” page 9, line 189*

4. Methodology 2.1: Can the search terms be clearly defined, with appropriate and consistent use of quotation marks, as represented in S2 attachment.

The search terms have now been clearly defined with appropriate and consistent use of quotation marks, as reflected in Section 2.1 of the methodology and the revised S2 attachment.

5. Methodology Figure 1: Ensure text has correct punctuation and capitalisation, and all lines are connecting to relevant boxes in the flow chart

Thanks for the observation, we have corrected this

6. Methodology 2.2: It would be clearer for the reader if eligibility and exclusion criteria were presented in dot point form. The comment that abstracts, conference proceedings.... grey literature are excluded, would be more clearly referenced as exclusion criteria.

We thank the reviewer for this helpful suggestion. The eligibility and exclusion criteria have now been reformatted into numbered points for clarity, and the exclusion of abstracts, conference proceedings, and grey literature is now explicitly presented under the exclusion criteria.

7. Methodology 2.3: Needs significant review of language, use of past/present tense, and sentence structure.

Thank you for your helpful feedback regarding the language, tense, and sentence structure in Section 2.3. We have carefully revised this section to improve clarity, ensure consistent use of past tense, and enhance readability.

8. Results: Needs significant review to improve clarity of expression, use of formal language and concise reporting.

Thanks for the comments. The results section has been thoroughly reviewed to ensure clarity in expression. We have integrated discussions into the results section which has increased the length of the section following review.

9. In particular, use of tables needs refining, consider formatting, use of language and overall presentation. E.g. Tables 3-6 are not clear what they are indicating and seem to use ticks across them for different reasons. Please consider representing this more clearly, such as with footnotes below the table to aid the reader.

Footnotes have been added to the tables

10. Subheads are also inconsistent, e.g. 3.5.1. speaks generally of enablers compared to 3.5.2-4 each represent a specific barrier.

Subheadings have been corrected, the enabler section is now labelled as 3.5 and the Barriers is 3.6

11. As the reporting of results appears combined with discussion points throughout, consider whether this section would be more accurately titled "Results/Discussion", or if the discussion points can be moved to the appropriate section.

Thanks for the comments. We have now integrated the discussion with results and now have a Results and Discussions section.

12. Discussion: As above, this section seems to repeat the main results. Consider whether this could be more concisely presented a discussion of overall themes (as it seems to be towards the second half of the section), or more clearly defined responses to the main research questions.

We have now revised the section to reflect implications rather than a repeat of the results. Consequently, we have renamed this section Conclusions and Implications to reflect this change. We have now moved components of discussion of results into the results section.

13. Attachment S3: There are only 5 studies represented in the Risk of Bias MMAT tool, yet the review included 6.

This has been corrected and now includes 6 studies

REVIEWER 1

Despite the significant findings from the review, the reporting of results appears inconsistent and hard to follow, leading to a weak discussion with clear conclusions and useful recommendations for future research and service design.

Thanks for the comments. The results section are organised in line with the research questions for readers to easily follow. We have also now integrated results and discussion for an integrated section. The Discussion section has now been renamed Conclusions and Implications which contains information related authors’ perspectives on the review as well as identifying areas for future research and intervention.

REVIEWER 2

Minor revisions are suggested below:

PROSPERO registration

Page 8 (line 176 mentions “Registration number: XXXXXX”). Suggestion: Add the true registrationnumber to complete the transparency of the protocol.

We have included it in the text as shown below

*“The review protocol was registered in the Prospective Register of Systematic Reviews (PROSPERO), registration number CRD42024561692” page 9, line 189*

Parental acceptability

Page 32 (lines 555–580 in section 3.6). Suggestion: This section notes that direct parentalperspectives were not captured, relying instead on inferred data. Explicitly stating this as alimitation in the conclusion or discussion would add important caution to this aspect of the paper.

We have included caution in the result and conclusion as shown below

*“While further research is needed on parental perspectives, reports suggest that engagement and acceptance of interventions were high*” Page 32*.*

“In addition, the review found little evidence regarding parental perspectives, fathers’ engagement and the effectiveness of interventions across diverse parent populations, such as disabled parents or ethnic minorities outside Indigenous groups.” page 37-38, line 692

Language clarity

Throughout the manuscript – but particularly noticeable in sections like the Abstract,

This is noted and has been corrected

Introductionand Results/Discussion. Suggestion: Minor grammatical tweaks and trimming of run-on sentenceswould enhance readability, especially for an international audience.

Thanks for the comments. The manuscript has been thoroughly proofread ensuring there is consistency throughout, avoiding double spaces, ensuring there is a space before citation, correcting minor grammatical errors and ensuring the use of the past tense in appropriate places in the methodology and results section.
