# Supplementary material for: Breaking the cycle: Systematic review of perinatal interventions for parents at risk of child removal
Source: PLoS One. 2025 Nov 26;20(11):e0337711. doi: 10.1371/journal.pone.0337711 (PMC12654914; doi:10.1371/journal.pone.0337711)
Supplement: Attachment — Submitted filename: Response_to_Reviewers_auresp_2.docx [file pone.0337711.s007.docx]

**Response to Reviewers**

**Reviewer Comments:**

1. Consistency in your use of USA or U.S/UK and United Kingdon, being sure to spell all acronyms out when first using them in the manuscript i.e. Page 4 Line 83 / Page 5 Line 94

Response: Corrected to “and Black families in the United States of America (USA) identified-

proceedings had previously had at least one child removed (16). Studies in the USA show ”

2. Consistency in spelling out the word for numbers ten and below, and using numerical figures for numbers greater than 10 e.g. Page 4 Line 73 and many instances throughout the manuscript

Response: Corrected to “Australia, infants (children under one year old) represent the largest proportion of child”

3. Page 4 - Check for consistency in capitalization for the word 'Black'

All changed

4. Page 4 Line 93 - Avoid starting the sentence with a numerical figure, unless writing it out full in text.

Response: Corrected to “Ninety two percent of Scottish mothers with older…”

5. Page 9 Line 191 - Please capitalise APA PsycInfo and PsycArticles correctly and ensure this is consistent with Figure 1

Response: Corrected to “We systematically searched six electronic databases (i.e., APA PsycINFO, PsycARTICLES, Scopus, PubMed, CINAHL, and Web of Science).

6. Page 12 Line 266 - be consistent with capitalising (or not) the first word in each dot point of the exclusion criteria

Response: Corrected to “were abstracts, conference proceedings, reviews and grey literature.”

7. Page 15 Line 320 - Please edit to read "None of the included studies reported on any interventions or engagement with fathers"

Response: Corrected to “None of the included studies reported on any intervention with fathers or engagement with fathers. “

8. Page 15 Line 326 - Please capitalise First Nations

Response: Corrected to “vulnerable Indigenous (First Nations) mothers”

9. Please spell out and capitalise proper nouns correctly, e.g. Page 15, Line 337 Australian Capital Territory; Page 16 Line 342 Parents under Pressure

Done

10. Page 16 Line 358 - Please reword 'till' to 'until' or 'to'

Response: Corrected to “Three interventions provided support up untill one year after birth

11. Page 17 Line 365 - Please correct the grammar in the final sentence and change question to 'questions'

Done

12. Please check all formatting is correct in Table 1, including consistent use of spaces between statistical results e.g. = % <; language is clear and grammatically correct e.g. Reference (32) child removal outcome

Response: Table is now corrected

13. Page 17 and other places in manuscript - be consistent with terminology infants rather than babies as previous noted

Response: All changed to infants

14. Please consider the overall formatting of Tables 2 and 3 and whether the columns could instead be rows and the rows be columns, to fit more clearly within the portrait frame

Response: Table is now corrected

15. Page 23 Line 420 - Please correct the word 'present' to 'presents'

Done

16. Page 26 Line 292 - Please write 'approaches' in the heading and first sentence. Please further check this paragraph for correct formatting

Done

17. Please capitalise the word Table on Page 27 Line 519

Done

18. Please include all studies in Table 4 or consider the usefulness of the table outside of describing the four common elements and four studies that reported on them.

Response: Table is now corrected to include all studies

19. Page 28 - Consider being more concise with your subheadings in Section 3.6. and removing the words 'As a barrier"

Response: 'As a barrier “is removed

20. Page 29 - please check formatting on this page and consistency of intext referencing

Response: formatting on page is now corrected

21. Page 29 - consider rewording the title of subheading 3.6.2. to "Fear of child removal and distrust of services"

Done

22. Page 29 Line 566-567 - Please improve the clarity and accuracy of this sentence using the word mothers twice

Done

23. Page 31 Section 3.2 - Please review and enhance the clarity of this first paragraph.

Done

24. Page 32 Line 616 - please change the word 'staffs'

Done

25. Page 32 Lines 626-627 - Please review and improve the wording of this statement to be clearer and accurate. E.g. While further research is needed on parental perspectives, for those studies that did address acceptability and engagement of interventions, reports were high (as displayed in Table 3).

Done

26. Please review wording and formatting in Table 6 and improve readability

Done

27. Page 36 Line 666 - Remove the word 'the' before 'infant'

Done

28. Page 37 - Please revise and improve the language in this first paragraph. The last sentence is very long and difficult to read.

Done

29. Page 37 Line 677 - Please clarify "intervention effectiveness in reducing infant removals"

Done

30. Page 37 Line 685 - Consider clarifying the "need for greater quality and strength of evidence on ...." and improving the wording of the following sentence cross Lines 686 – 689

Done. Changed into “Although there are evaluation reports that address recurrent care proceedings and infant removal, only six peer-reviewed studies met the inclusion criteria for this review”

31. Page 38 Line 697 - Please consider why only the UK as the review has also highlighted need related to particular groups in other countries i.e. First Nations parents in Australia

Changed to “and especially in the context where rates of recurrent care proceedings still persist”
